# Fucoidan inhibits the migration and proliferation of HT-29 human colon cancer cells via the phosphoinositide-3 kinase/Akt/mechanistic target of rapamycin pathways

**DOI:** 10.3892/mmr.2015.3804

**Published:** 2015-05-21

**Authors:** YONG-SEOK HAN, JUN HEE LEE, SANG HUN LEE

**Affiliations:** 1 Laboratory for Medical Science Research Institute, Soonchunhyang University, Seoul Hospital, Seoul 336-745, Republic of Korea; 2 Department of Physiology, Laboratory for Vascular Medicine and Stem Cell Biology, Medical Research Institute, School of Medicine, Pusan National University, Yangsan, Gyeongsangnam-do 626-870, Republic of Korea

**Keywords:** fucoidan, phosphoinositide-3 kinase/Akt, proliferation, migration, sphere formation

## Abstract

Fucoidan, a sulfated polysaccharide, has a variety of biological activities, including anti-cancer, anti-angiogenic and anti-inflammatory effects. However, the underlying mechanisms of fucoidan as an anti-cancer agent remain to be elucidated. The present study examined the anti-cancer effect of fucoidan on HT-29 human colon cancer cells. The cell growth of HT29 cells was significantly decreased following treatment with fucoidan (200 *μ*g/ml). In addition, fucoidan inhibited the migration of HT-29 cells by decreasing the expression levels of matrix metalloproteinase-2 in a dose-dependent manner (0–200 *μ*g/ml). The underlying mechanism of these inhibitory effects included the downregulation of phosphoinositide 3-kinase (PI3K)/Akt/mammalian target of rapamycin (mTOR) by treatment with fucoidan. Furthermore, fucoidan increased the expression of cleaved caspase-3 and decreased cancer sphere formation. The present study suggested that fucoidan exerts an anti-cancer effect on HT-29 human colon cancer cells by downregulating the PI3K-Akt-mTOR signaling pathway. Therefore, fucoidan may be a potential therapeutic reagent against the growth of human colon cancer cells.

## Introduction

Colon cancer is one of the most prevalent types of cancer in the United States and is the second most frequent cause of cancer-associated mortality (1). In addition, the worldwide incidence rates of colorectal cancer have been increasing steadily in previous years. Although early-stage colorectal cancer can be successfully treated by surgery, advanced-stage colon cancer frequently recurs and becomes fatal, even in patients receiving combination chemotherapy (2). Chemotherapeutic agents, including cisplatin, are routinely used in the treatment of advanced-stage colon cancer; however, they provide only minimal survival benefits as a result of several factors: Drug resistance, side effects and toxicity (3,4). Previously, the development of cancer chemoprevention protocols using natural or synthetic agents, which prevent or suppress the progression to invasive cancer, have been recognized as a field with enormous potential to reduce the cancer burden (5). Therefore, there is an urgent requirement for novel chemopreventive agents with minimal or no side effects and toxicities. In previous years, bioactive compounds derived from natural sources have become the focus of a substantial amount of attention from researchers seeking to develop chemopreventive agents, primarily due to the potential cancer-preventive and/or therapeutic activities of several of these compounds at non-toxic levels. However, continued research into the mechanism of action of such compounds is required.

Fucoidan is a sulfated polysaccharide located in the cell wall matrix of brown seaweeds, including *Ascophyllum nodosum, Cladosiphon okamuranus, Ecklonia kurome, Fucus evanescens, Fucus vesiculosus, Hizikia fusiforme, Laminaria angustata* and *Undaria pinnatifida* (6–8). Structurally, fucoidan is a heparin-like molecule with a substantial percentage of l-fucose and sulfated ester groups, as well as small quantities of d-xylose, d-galactose, d-mammose and glucuronic acid (9). Among the several analogues of fucoidan, the predominant form is isolated from *Undaria pinnatifida* and is described as a sulfated galactofucan (10). Fucoidan has various biological activities, including anti-cancer (11), anti-inflammatory, anti-angiogenic (12), anti-coagulant (13) and anti-human immunodeficiency virus (14) activities. In previous *in vivo* studies performed using xenograft models, fucoidan was reported to suppress the growth of Ehrlich ascites carcinoma (15,16) and Lewis lung adenocarcinoma (17), and was also demonstrated to inhibit the metastasis of Lewis lung adenocarcinoma (17) and 13762 MAT rat mammary adenocarcinoma (18). These findings demonstrated that fucoidan inhibits the growth of human non-small-cell bronchopulmonary carcinoma (NSCLS-N6) cells (19) and human lymphoma HS-Sultan cells (11), and also inhibits the invasion of HT1080 human fibrosarcoma cells and the angiogenic activity of HeLa human uterine carcinoma cells (20).

Fucoidan cannot be hydrolyzed by digestive enzymes in the human small intestine (21) and therefore, the consumption of this compound can result in an increase in the concentration of luminal fucoidan within the large intestine. Thus, fucoidan may prove to be an excellent candidate for the prevention of colon carcinogenesis, provided that it exerts cancer-preventive effects in the colon. However, its inhibitory mechanism on colon cancer proliferation and metastasis remains to be elucidated. In the present study, the effect of fucoidan on the migration and proliferation of HT-29 human colon cancer cells, and its underlying anti-cancer mechanisms of action were investigated.

## Materials and methods

### Preparation of fucoidan

Fuciodan extract from the seaweed *Fucus vesiculous* was obtained from Sigma-Aldrich (St. Louis, MO, USA). Fucoidan powder was dissolved in phosphate-buffered saline (Gibco Life Technologies, Carlsbad, CA, USA), sterilized by filtration through a 0.45-*μ*m pore filter (Sartorius Biotech GmbH, Göttingen, Germany) and stored as fucoidan extract (20 mg/ml) at 4°C until use.

### Cell cultures

The human HT-29 colon cancer cell line was obtained from the American Type Culture Collection (Manassas, VA, USA). The cells were maintained in Dulbecco's modified Eagle's medium (4.5 g/l glucose), supplemented with 10% fetal calf serum, l-glutamine and antibiotics (Biological Industries, Beit Haemek, Israel) at 37°C with 5% CO_2_ in a humidified incubator.

### Cell viability assay

Exponentially growing colon cancer cells in 96-well plates (5,000 cells/well) were sub-confluently incubated with fucoidan (0, 50, 100 and 200 *μ*g/ml) for various durations (24 and 48 h). Cell viability was determined using a modified version of the MTT assay (Promega, Madison, WI, USA), which was based on the conversion of the tetrazolium salt of MTT to the formazan product by mitochondrial dehydrogenase. A total of 10 *μ*l MTT solution was added to each well and incubated for 4 h at 37°C. The color was extracted with dimethyl sulfoxide (Sigma-Aldrich) at 37°C for 20 min. The formazan product was quantified by measuring the absorbance of the reaction at 570 nm using a microplate reader (Infinite F50; Tecan, Männedorf, Switzerland).

### Wound-healing migration assay

The HT-29 cells were seeded onto six-well plates (25×10^4^ cells/well) and grown to 90% confluence in 2 ml growth medium. The cell monolayers were damaged using a 2 mm-wide tip to generate a line-shaped wound. The cells were subsequently treated with fucoidan (0, 50, 100 and 200 *μ*g/ml) for 48 h at 37°C. The cells were allowed to migrate and images were captured by an inverted microscope (IX71; Olympus, Tokyo, Japan).

### Tumor sphere culture

To generate tumor spheres, the colon cancer cells (200 cells/ml) were seeded into siliconized spinner flasks (Bellco, Vineland, NJ, USA), followed by agitation at 70 rpm for three days. Spinner flasks were siliconized by the application of Sigma coat (Sigma-Aldrich), followed by drying for a minimum of 24 h. The cells were cultured with growth medium. Spheroids formed at day three and images of the spheres were captured by an inverted microscope (IX71; Olympus).

### Western blot analysis

The total protein was extracted using radioimmunoprecipitation lysis buffer (Thermo Fisher Scientific, Waltham, MA, USA). The cell lysates were separated via SDS-PAGE (Bio-Rad Laboratories, Inc., Hercules, CA, USA) and were transferred onto a polyvinylidene fluoride membrane (Millipore, Billerica, MA, USA). The membranes were blocked with 5% non-fat milk (Wako Pure Chemical Industries, Ltd., Osaka, Japan) and were subsequently incubated with the appropriate primary antibodies: Mouse monoclonal cyclin D1 (1:1,000; cat. no. sc-20044; Santa Cruz Biotechnology, Inc., Dallas, TX, USA), mouse monoclonal cyclin E (1:1,000; cat. no. sc-377100; Santa Cruz Biotechnology, Inc.), rabbit polyclonal CDK2 (1:1,000; cat. no. sc-748; Santa Cruz Biotechnology, Inc.), mouse monoclonal CDK4 (1:1,000; cat. no. sc-56277; Santa Cruz Biotechnology, Inc.), mouse monoclonal matrix metalloproteinase 2 (MMP 2; 1:1,000; cat. no. sc-13594; Santa Cruz Biotechnology, Inc.), rabbit monoclonal p-Akt (1:1,000–1:2,000; cat. no. OMA1-03061; Pierce Biotechnology, Inc., Rockford, IL, USA), rabbit poly-clonal p-mammalian target of rapamycin (mTOR; 1:1,000; cat. no. sc-101738;Santa Cruz Biotechnology, Inc.), mouse monoclonal p-p70s6k (1:1,000; cat. no. sc-8416; Santa Cruz Biotechnology, Inc.), rabbit cleaved caspase-3 (1:1,000; cat. no. 9664; Cell Signaling Technology, Danvers, MA, USA) and mouse monoclonal β-actin (1:3,000; cat. no. sc-47778; Santa Cruz Biotechnology, Inc.) in Tris-buffered saline in 0.1% Tween-20 (TBST) and incubated overnight at 4°C. The membranes were subsequently washed three times with TBST, incubated at 4°C overnight with goat anti-mouse (1:10,000; cat. no. sc-2005; Santa Cruz Biotechnology, Inc.) and goat anti-rabbit (1:10,000; cat. no. sc-2004; Santa Cruz Biotechnology, Inc.) secondary antibodies. The bands were visualized by enhanced chemiluminescence reagents (Amersham Biosciences, Uppsala, Sweden). Quantification of band intensity was performed using TINA 2.0 (Raytest, Straubenhardt, Germany) and normalized against the intensity of β-actin.

### Statistical analysis

Values are expressed as the mean ± standard error of the mean. All experiments were analyzed by Student's t-test. P<0.05 was considered to indicate a statistically significant difference. All data were analyzed using Sigma plot 8.0 software (Systat Software Inc., San Jose, CA, USA).

## Results

### Fucoidan inhibits the proliferation of human HT-29 colon cancer cells

**Figure 1:**
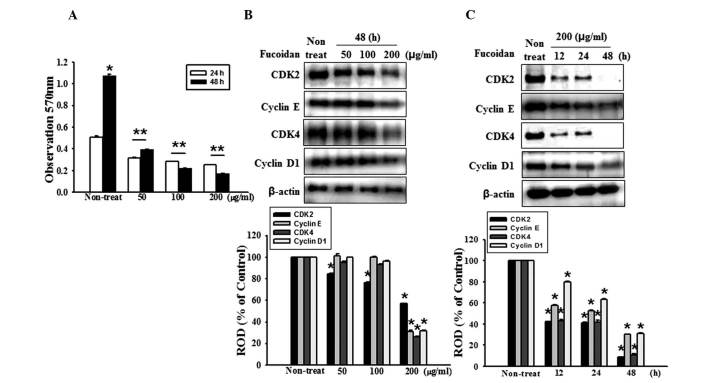
Fucoidan inhibits the proliferation of HT-29 cells. (A) HT-29 cell proliferation was measured using an MTT assay. Values are expressed as the mean ± standard error of the mean from three independent experiments (^*^P<0.05, ^**^P<0.01, vs. 48 h fucoidan treatment). (B) HT-29 cells were incubated for 48 h with various concentrations of fucoidan (0, 50, 100 and 200 *μ*g/ml) and the expression levels of CDK2, cyclin E, CDK4 and cyclin D1 were assessed by western blotting. (C) HT-29 cells were treated with fucoidan for different durations (0–48 h). The expression levels of CDK 2, cyclin E, CDK4 and cyclin D1 were assessed by western blotting. Values are expressed as the mean ± sandard error of the mean of four independent experiments for each condition, as determined from densitometry against to β-actin (^*^P<0.05, vs. non-treated cells). ROD, relative optical density.

The effects of various fucoidan concentrations (50, 100, 200 *μ*g/ml) on the growth of HT-29 cells were initially assessed by measuring the viable cell numbers via the MTT assay. Fucoidan reduced the number of viable HT-29 cells in a dose- and time-dependent manner (Fig. 1A). In addition, the present study assessed whether fucoidan affects the expression levels of the cell cycle regulatory proteins, cyclin D1, cyclin E, CDK2 and CDK4. These proteins were demonstrated to be maximally decreased following 48 h of treatment with 200 *μ*g/ml fucoidan (Fig. 1B and C). Collectively, these data demonstrated that fucoidan may suppress the proliferation of colon cancer cells.

### Fucoidan inhibits the expression of MMP-2 and the migration of HT-29 cells

**Figure 2:**
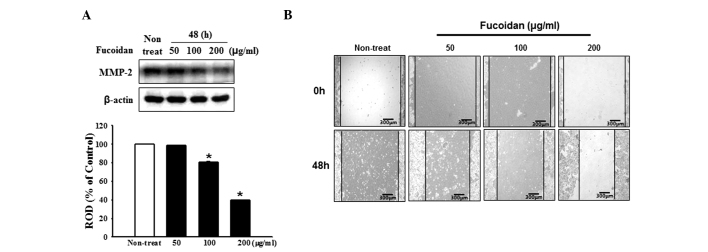
Fucoidan inhibits the migration of HT-29 cells. (A) HT-29 cells were incubated for 48 h with various concentrations of fucoidan (0–200 *μ*g/ml) and the expression of MMP-2 was assessed by western blotting. The protein expression levels were quantified and the values are expressed as the mean ± standard error of the mean of five independent experiments, as determined by densitometry against β-actin (^*^P<0.05, vs. non-treated cells). (B) The HT-29 cells were seeded onto six-well culture plates and grown to 90% confluence in media containing 10% fetal bovine serum. The cells were scratched with a scraper and subsequently treated with fucoidan (0, 50, 100 and 200 *μ*g/ml). The images are representative of four independent experiments (top lane; incubation for 0 h, bottom lane; incubation for 48 h). MMP, matrix metalloproteinase; ROD, relative optical density.

The degradation of the extracellular matrix (ECM) is crucial for cellular migration and invasion, indicating the inevitable involvement of matrix-degrading proteinases. Therefore, the present study examined the effect of fucoidan on the expression of MMP-2, a key molecule involved in ECM degradation, by western blotting. The expression of MMP-2 was gradually reduced in response to an increase in fucoidan concentration (Fig. 2A). Cell migration is a measure of the metastatic potential of cancer cells; therefore, the influence of fucoidan on cell migration was investigated using a wound-healing assay. The HT-29 cells treated with fucoidan demonstrated a reduction in cell migration (Fig. 2B). Collectively, these data demonstrated that fucoidan suppressed the migratory properties of human colon cancer cells.

### Fucoidan suppresses the signaling of PI3K

**Figure 3:**
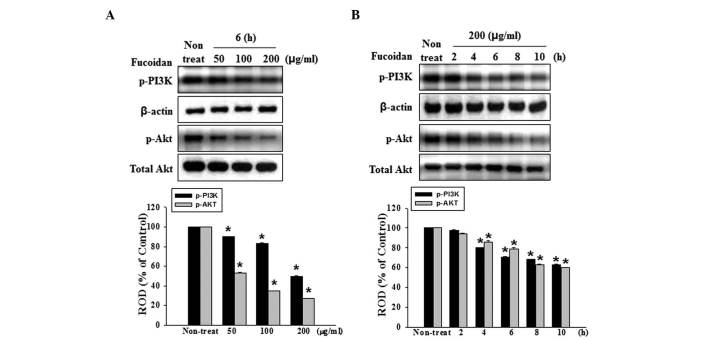
Dose- and time-dependent effects of fucoidan on PI3K and Akt in HT-29 cells. (A) HT-29 cells were incubated for 6 h with various concentrations of fucoidan (0–200 *μ*g/ml) and the phosphorylation of PI3K and Akt was detected by western blotting. (B) HT-29 cells were treated with fucoidan (200 *μ*g/ml) for 0, 2, 4, 6, 8 and 10 h and the phosphorylation of PI3K and Akt was detected by western blotting. The protein expression levels were quantified and vallues are expressed as the mean ± standard error of the mean of five experiments, as determined by densitometry against β-actin (^*^P<0.05, vs. non-treated cells). ROD, relative optical density; p-PI3K, phosphorylated phosphoinositide-3 kinase.

The above findings have demonstrated that fucoidan significantly inhibits the proliferation and migration of HT-29 cells, and also reduced the expression of MMP-2 in HT-29 cells. However, the signaling mechanisms responsible for fucoidan on proliferation and migration remain to be elucidated. PI3K/Akt has been suggested as a key pathway involved in the regulation of proliferation and migration. The present study revealed that fucoidan markedly inhibited the phospholylation of PI3K and its downstream target, Akt, in a dose- and time-dependent manner (Fig. 3A and B). Based on these results, fucoidan potently inhibited the proliferation and migration of human colon cancer cells, possibly by suppressing the PI3K/Akt pathway.

### Fucoidan suppresses mTOR signaling

**Figure 4:**
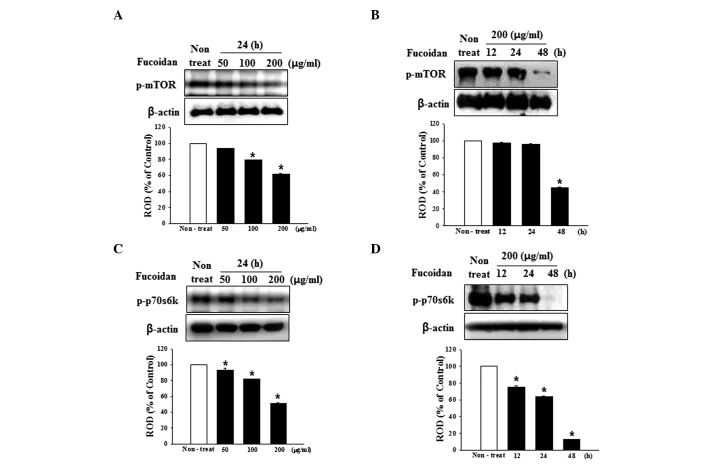
Dose- and time-dependent effects of fucoidan on mTOR in HT-29 cells. (A) HT-29 cells were incubated for 24 h with various concentrations of fucoidan (0–200 *μ*g/ml) and the phosphorylation of mTOR was detected by western blotting. (B) HT-29 cells were treated with fucoidan (200 *μ*g/ml) for 0, 12, 24 and 48 h and the phosphorylation of mTOR was detected by western blotting. (C) HT-29 cells were incubated for 24 h with various concentrations of fucoidan (0–200 *μ*g/ml) and the phosphorylation of p70S6K was detected by western blotting. (D) HT-29 cells were treated with fucoidan (200 *μ*g/ml) for 0, 12, 24 and 48 h and the phosphorylation of p70S6K was detected by western blotting. The protein expression levels were quantified and values are expressed as the mean ± standard error of the mean of five experiments, as determined by densitometry against β-actin (^*^P<0.05, vs. non-treated cells). ROD, relative optical density; p-mTOR, phosphorylated mechanistic target of rapamycin.

Based on the above finding that fucoidan suppresses the PI3K-Akt pathway in human HT-29 colon cancer cells, whether fucoidan modulates mTOR and its downstream signaling molecules was investigated. As shown in Fig. 4A and B, fucoidan inhibited the phospholylation of mTOR in a dose- and time-dependent manner. In addition, the phosphorylation of p70S6K, an immediate downstream target of mTOR and an indicator of mTOR activity, was also significantly suppressed (Fig. 4C and D).

### Fucoidan increases the activation of caspases

**Figure 5:**
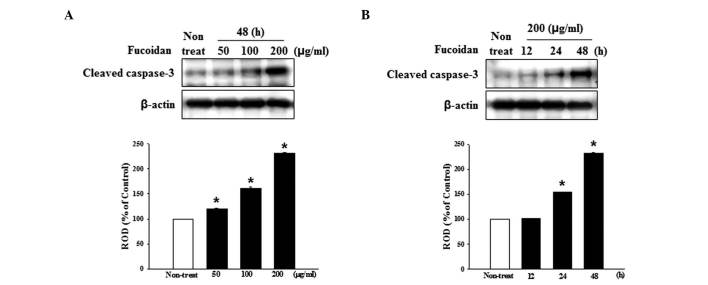
Fucoidan induces the apoptosis of HT-29 cells. (A) HT-29 cells were incubated for 48 h with various concentrations of fucoidan (0–200 *μ*g/ml) and the expression of cleaved caspase-3 was detected by western blotting. (B) HT-29 cells were treated with fucoidan (200 *μ*g/ml) for 0, 12, 24 and 48 h and the expression of cleaved caspase-3 was detected by western blotting. The protein expression levels were quantified and values are expressed as the mean ± standard error of the mean of five experiments, as determined by densitometry against β-actin (^*^P<0.05, vs. non-treated cells). ROD, relative optical density.

Caspases are central effectors of apoptosis. To examine the mechanism of fucoidan-induced apoptosis, western blotting was used to detect anti-cleaved caspase-3, which detects the cleaved forms of the enzymes to determine whether or not fucoidan activated caspases. Treatment with fucoidan increased the expression of cleaved caspase-3 in a dose- and time-dependent manner (Fig. 5A and B).

### Fucoidan inhibits cancer sphere formation

**Figure 6:**
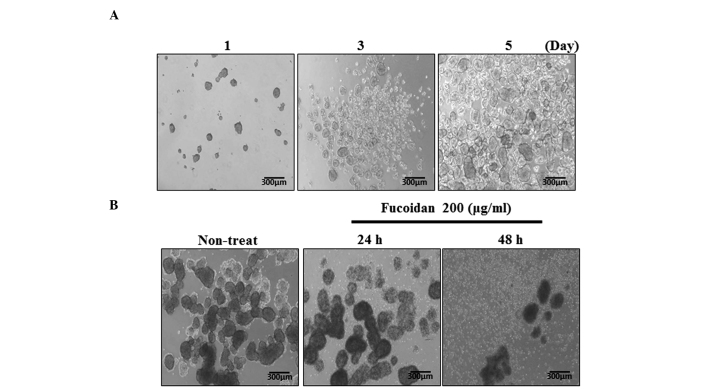
Fucoidan inhibits cancer sphere formation. (A) HT-29 cells were seeded at 500 cells/well of a six-well culture plate. Tumor spheres were formed five days following seeding. (B) The inhibition effect of fucoidan induced cancer sphere formation. The images shown are representative of four independent experiments.

In order to generate spheroid cells, HT-29 cells were enzymatically dissociated and inoculated on ultra-low attachment culture plates in serum-free medium. As is often the case for HT-29 cells, the majority survived and generated floating spherical colonies following 3–5 days in culture (Fig. 6A). To investigate the cancer-sphere-formation capacity, the HT-29 cells were treated with fucoidan during sphere formation. The results demonstrated that sphere formation by the HT-29 cells was inhibited by fucoidan in a time-dependent manner (Fig. 6B). At 200 *μ*g/ml fucoidan, the efficiency of cancer sphere formation was reduced.

## Discussion

Given the high mortality rate as a result of colon cancer and the significant morbidity, apparent toxicity and poor response rates of current chemotherapeutic regimens, there has been a big push to identify novel therapeutic modalities with fewer toxicity profiles. The PI3K/Akt/mTOR signaling axis is critical in the proliferation, resistance to apoptosis, angiogenesis and metastasis, and is central to the development and maintenance of colorectal cancer cells (21–23). Accordingly, inhibition of the activity and expression of the Akt pathway may lead to the development of effective therapy in patients with colon cancer. Although numerous studies have made great efforts to develop potential anti-cancer agents inhibiting the Akt pathway, the majority of clinical trials remain to be successfully completed (24).

In search of efficacious tumor suppressors, a wide variety of natural products and the pharmacologically active components of plants are promising candidates. These natural products have several advantages over synthetic chemicals with regards to side effects and pharmacophore diversity. Certain products from various natural resources have previously been characterized as potential therapeutics with anti-cancer activity (22). Among them, fucoidan has been demonstrated to possess anti-proliferative and cytotoxic effects on MCF-7 breast cancer cells; however, not on human mammary epithelial cells (23). Previously, fucoidan was demonstrated to inhibit metastasis by suppressing MMP-2/29 and reducing the expression and secretion of a vascular endothelial growth factor (14). However, the underlying mechanism of its inhibition of cancer cell proliferation and metastasis remains to be elucidated. The present study partly identified, for the first time, to the best of our knowledge, the underlying molecular mechanism of the anti-proliferative and anti-metastatic effects of fucoidan.

Cell viability was assessed using an MTT assay in the absence or presence of various concentrations of fucoidan. Fucoidan inhibited cell growth, the expression of MMP-2 and cell migration capacity at 200 *μ*g/ml. Therefore, the anti-proliferative and anti-migratory effects of fucoidan observed in the present study were dependent on its cancer-preventive effects.

The Akt signaling pathway is known to regulate the development and progression of various types of tumor (24–26). There is an accumulating number of studies demonstrating the importance of the Akt signaling pathway in the inhibition of cell growth (24–27). Previous studies have revealed a decreased activation of Akt in growth-retarded tumor cells (24–27). Several previous studies have also demonstrated that targeting of the PI3K/Akt signaling pathway with anti-sense small interfering (si)RNA or small molecule inhibitors results in the downregulation of tumor invasion and tumorigenesis in malignant cancer cells (28,29). The results of the present study demonstrated that fucoidan inhibited the phosphorylation of PI3K/Akt in a time- and dose-dependent manner. PI3K and Akt are well-known upstream regulators of the mTOR signaling pathway in mammalian cells. As evidence of this hypothesis, siRNA-mediated gene silencing of PI3K and Akt inhibited the activation of p70S6K1, a downstream target of mTOR, and subsequently led to the suppression of migration, invasion and proliferation (30). In the present study, fucoidan significantly decreased the phosphorylation of mTOR and p70S6K1. In addition, since previous studies indicated that spheroid cells were more resistant to chemotherapeutic drugs (31–34), the present study assessed the sphere formation capacity of HT-29 cells during treatment with fucoidan. The results revealed that fucoidan inhibited sphere formation in a time-dependent manner. Fucoidan concentrations of 200 *μ*g/ml caused significant inhibition of sphere formation in HT-29 cells.

In conclusion, the present study demonstrated for the first time, to the best of our knowledge, that fucoidan inhibited cell growth, migration and sphere formation by suppressing the PI3K/Akt/mTOR pathway and reducing the expression of MMP-2 in human HT-29 colon cancer cells. *In vivo* and clinical investigations are required for the development of fucoidan as a novel therapeutic agent and alternative remedy in patients with cancer. By any measure, fucoidan may be a promising candidate as an efficacious anti-cancer reagent with minimal side effects in normal cells.
